# Intelligent Evacuation Route Planning Algorithm Based on Maximum Flow

**DOI:** 10.3390/ijerph19137865

**Published:** 2022-06-27

**Authors:** Li Liu, Huan Jin, Yangguang Liu, Xiaomin Zhang

**Affiliations:** 1 College of Digital Technology and Engineering, Ningbo University of Finance and Economics, Ningbo 315175, China; liuli6883@nbufe.edu.cn (L.L.); zhangxiaomin@nbufe.edu.cn (X.Z.); 2 Department of Computer Science, University of Nottingham Ningbo China, Ningbo 315100, China; 3 College of Finance and Information, Ningbo University of Finance and Economics, Ningbo 315175, China

**Keywords:** evacuation routing, network flow algorithm, artificial intelligence, route planning

## Abstract

This paper focuses on the problem of intelligent evacuation route planning for emergencies, including natural and human resource disasters and epidemic disasters, such as the COVID-19 pandemic. The goal of this study was to quickly generate an evacuation route for a community for victims to be evacuated to safe areas as soon as possible. The evacuation route planning problem needs to determine appropriate routes and allocate a specific number of victims to each route. This paper formulates the problem as a maximum flow problem and proposes a binary search algorithm based on a maximum flow algorithm, which is an intelligent optimization evacuation route planning algorithm for the community. Furthermore, the formulation is a nonlinear optimization problem because each route’s suggested evacuation time is a convex nonlinear function of the number of victims assigned to that route. Finally, numerical examples and Matlab simulations demonstrate not only the algorithm’s effectiveness, but also that the algorithm has low complexity and high precision. The study’s findings offer a practical solution for nonlinear models of evacuation route planning, which will be widely used in human society and robot path planning schemes.

## 1. Introduction

Evacuation is a key task in the emergency management response stage. For any public safety incident, especially in the global prevention and control of COVID-19, the evacuation of victims and potential victims from the victim site to a safe area has become a top priority [1]. In the process of personnel evacuation, people need to make decisions or deal with a series of subproblems as a key strategy in the emergency response stage, including predicting the evolution of disaster impacts, dividing evacuation areas, issuing evacuation instructions, estimating evacuation traffic needs, and determining evacuation plans. Then, evacuees must be guided to adjacent evacuation routes, and traffic signals must be updated to effectively evacuate traffic [2]. According to the actual situation, people determine the evacuation plan. That is to say, selecting the appropriate means of transportation and distributing the number of evacuees to each evacuation route according to the traffic conditions is the core issue of the evacuation problem. An effective evacuation plan reduces disaster losses, especially the occurrence of mass casualties [3].

The purpose of an evacuation plan is to establish procedures for orderly and coordinated evacuation routes for a community. This can be applied to different types of emergencies that may require evacuation from homes to shelters or quarantined areas. For natural and human resource disaster emergencies such as typhoons, floods, volcanoes, tsunamis, tornadoes, and home fires, evacuation plans can be applied to evacuate people from homes to shelters. For epidemic disasters, such as COVID-19, the proposed plan can be applied to evacuate affected people from homes to quarantined areas in a timely manner. For different communities, it is essential to identify the common hazards that may occur and identify appropriate evacuation routes.

Regional evacuation refers to the process of moving people from a dangerous area to a safe area within a certain period. The study of regional evacuation involves the division of evacuation areas, the estimation of evacuation traffic needs, and the formulation of evacuation plans [4,5,6]. Community evacuation refers to the problem of evacuating the community population from a hazardous area to a safe area, which is a type of regional evacuation. With the increases in human resource and natural disasters, the government has established many shelters for various disasters. The primary purpose is to quickly transfer the disaster victims in the endangered area to the shelter when an emergency breaks out, to reduce the losses caused by the disaster [7]. In this paper, we formulate the community-based evacuation route planning problem as a maximum flow problem, which is a classical network flow problem. This formulation will determine the best evacuation routes from communities to shelters, and assign a specific number of victims to each route such that all victims can be transferred to destination areas. The main distinction of our model from the classical maximum flow formulation is that our model is a nonlinear model where each route’s suggested evacuation time is a convex nonlinear function of the number of victims assigned to that route. This nonlinearity raises challenges for solving the model. We propose a binary search algorithm based on the classical maximum flow solution approach, which can quickly generate solutions for the proposed model.

Emergency evacuation research first appeared in the 1960s in the form of proposing and discussing the concept and framework of emergency evacuation [8]. Later, the problem of emergency evacuation attracted the attention of many researchers. The main research methods are divided into micromodel research and macromodel research. Micromodels focus on the evacuated individuals’ behavior, psychology, and mutual influence. The influences of these factors on selecting evacuation paths and evaluating evacuation effectiveness is analyzed. The main microscopic models are the probability model, cellular automata model, social force model, and evacuation simulation model. The macromodel treats the people to be evacuated as a group and uses the optimization model method to solve the lower bound of the evacuation time for the evacuation path planning problem, and plans the entire evacuation route and plan from a macro perspective.

In this paper, the research on emergency evacuation path planning is based on the macromodel algorithm. A nonlinear model is established, and a polynomial time approximation algorithm is given, assuming no crossover between different paths and considering the nonlinear relationship between crowd density and evacuation speed. The main contributions of the paper are as follows. (1) We consider the evacuated people as a group and formulate the problem as an optimization model. (2) We assume no mutual influence between any two roads; the traffic conditions on one road do not affect those of another. The approximation algorithm based on network flow is provided to solve the model. (3) We theoretically analyze the complexity of the proposed algorithm, and simulation experiments prove the algorithm’s effectiveness.

## 2. Related Works

### 2.1. Macromodel of the Evacuation Path Problem

Macromodel research can study the model and algorithm of evacuation path planning. A good evacuation path planning model and algorithm can give a better evacuation planning scheme to effectively shorten the evacuation time. Hamacher [9] presented a detailed review of the macromodels of evacuation problems and introduced several models based on network flow. For example, minimum turnstile cost dynamic network flow models are used to estimate the average evacuation time of each evacuation individual; maximum dynamic flows and universally maximum dynamic flow models are used to calculate the maximum number of people evacuated within a given time limit; the fastest flow method (QFM) is used to estimate the minimum time to evacuate a certain number of people [10]. Most of these models assume that travel time is a fixed constant. If the nonlinear relationship between crowd density and evacuation time is considered, the complexity of the model will be greatly increased [11]. In this paper, the method to deal with the nonlinear relation replaces the nonlinear relation with an approximately linear function.

From the point of view of solving methods and algorithms of the macro evacuation model, it can be divided into a polynomial algorithm based on linear programming to solve the optimal evacuation scheme and various heuristic algorithms based on nonlinear programming [12,13,14,15]. The first method mainly uses the network flow method, especially the dynamic network flow method, to give the polynomial algorithm for various evacuation problems. A dynamic network [16] is defined on a directed graph G=(V, E), in which vertex set *V* contains source point sources, endpoint sinks, and a medium point set. Every arc (x, y)∈E has a non-negative capacity uxy and a non-negative transmission time $u_{xy}$. The two classical dynamic network flow problems are the maximum dynamic flow problem (MDFP) and the quick flow problem (QFP). MDFP refers to the dynamic flow of sinks to the endpoint under a given time limit T. QFP refers to moving a predetermined amount of traffic from a source point to an endpoint [17]. Burkard [18] presented the first strong polynomial algorithm for the QFP problem in the case of a single source point and single endpoint. Hoppe and Tardos [19] gave a polynomial algorithm for the evacuation problem with a fixed number of source points and endpoints. For a multi-source point and multi-endpoint QFP evacuation problem, they first used the binary search method to give an upper bound of optimal time T. Then, a polynomial algorithm of QFP through multi-source multi-end sinks was given by the ellipsoid method [20].

In addition to the dynamic network flow method, many studies use classical network flow methods, such as the algorithm of the least short circuit, least cost flow, and maximum flow to solve the evacuation path optimization problem. Dunn and Newton [21] used the maximum flow method to solve the problem of evacuation path allocation, aiming to transfer the evacuation crowd to the optimal path as far as possible within the capacity of the road network from the dangerous area to the safe area. Yamada [22] applied the least cost flow problem to allocate evacuation traffic paths and proposed the shortest evacuation plan (SEP), aiming at minimizing the total journeys of all the people to be evacuated to the designated shelter. Therefore, according to this optimization goal, each vehicle in the traffic network will choose the exit of the area nearest to it for evacuation. However, in a complex traffic network, such allocation rules can easily cause traffic congestion. In the lane-based evacuation network flow model proposed by Cova [23], the extended minimum cost flow model was established to prevent intersection conflicts and limit intersections while minimizing the total travel distance. Finally, the model outputs the possible road map of each intersection.

In summary, most researchers describe the evacuation problem as a network flow graph G(V, E) with nodes and edges with capacity constraints, in which some nodes are called source points, and each source point has a certain number of evacuees; and some nodes are called endpoints, and each endpoint can accommodate a certain number of evacuees. The edges in the figure represent evacuation paths. The evacuation problem is to evacuate all the people to the destination by allocating a reasonable number of people to each evacuation path to minimize the total evacuation time. The advantage of using the network flow theory to solve the problem is that the evacuation problem is transformed into a classic network flow problem and can be solved using the mature polynomial network flow algorithm, which is convenient and effective. However, the network flow problem can generally only solve the linear programming problem. The abovementioned articles can only solve linear problems; that is, the possible nonlinear relationship between crowd density and evacuation time cannot be considered in the model. Therefore, the model cannot reflect some evacuation traffic phenomena, such as slowing down due to traffic congestion.

### 2.2. Nonlinear Evacuation Planning

The nonlinear programming of the evacuation problem mainly considers the nonlinear relationship between crowd density and evacuation speed. At present, there is no polynomial algorithm for solving the nonlinear programming of evacuation problems, only a variety of heuristic algorithms. Pursals [24] considered building evacuation when crowd movement speed was affected by crowd density. First of all, referring to Nelson and McLennan’s analysis of crowd movement speed in an emergency, they gave the convex function relation between crowd evacuation time and crowd number at each exit [25]. This function is related to the length and width of the exit, the area occupied by the crowd before entering the exit, and the walking speed of the crowd under normal conditions. Then, the inverse function is the function of the time required to evacuate a specific number of people. In this paper, the inverse function of the evacuation function of all exits is summed, and the functional relationship between the total number of evacuations and evacuation time is obtained. Therefore, given the total number of people evacuated, K, the time required to evacuate them can be found by the function, M. Meanwhile, Carey [26] used a piecewise linear function to approximate the nonlinear relationship between evacuation time and the number of people and established a linear model to solve it.

As mentioned before, there are two types of methods for solving the evacuation problem in the literature. One is to model the problem as nonlinear programming and design a heuristic algorithm to solve it. The other is to model the problem as a network flow problem. The first type of method generally obtains an approximate solution, and the approximation ratio is not guaranteed. The advantage of the second method is that the evacuation problem is transformed into a classic network flow problem, and can be solved by using the mature polynomial network flow algorithm, which is convenient and effective. In this paper, we adopted the second method, and the nonlinear evacuation time with respect to the crowd density is considered in the model.

## 3. Materials and Methods

### 3.1. Community Evacuation Problem

The problem is how to transfer everyone that needs to be evacuated to designated shelters as soon as possible within the limited traffic network [27]. There is a nonlinear function relationship between crowd movement speed and crowd density. Furthermore, since crowd movement speed will slow down with increased density, the functional relationship between crowd movement speed and crowd density is a convex function [28,29].

**Figure 1:**
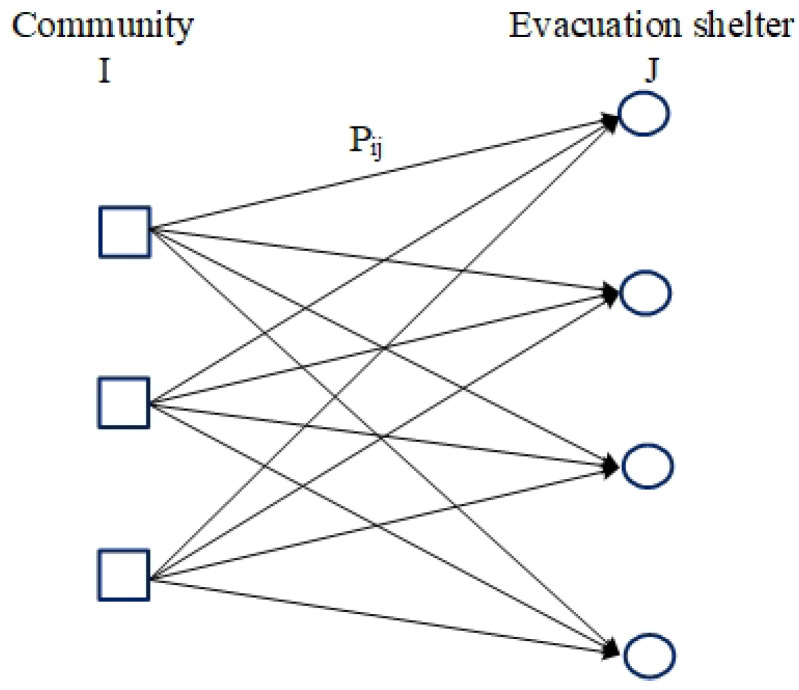
Schematic diagram of community evacuation route problem.

To sum up, this problem can be described as evacuating the population of *M* communities to *N* shelters, as shown in Figure 1. The problem includes the number of people to be evacuated in each community, the capacity limit of each shelter, the nonlinear function between evacuation time and the number of people evacuated on each path, and the values of parameters on each path from the community to the shelter (e.g., road length, road width, and intersection area). The fastest evacuation plan is to transfer all evacuated people in the community to the shelter—that is, the number of evacuees allocated to each road. Our goal is to minimize the time it takes to evacuate all communities to emergency shelters.

The sets $I=\left\{1,\;2,\;\ldots ,\;m\right\}$ and $J=\left\{1,\;2,\;\ldots ,\;n\right\}$ are used to denote the collections of communities and shelters, respectively. $b_{I}$ represents the number of people in community *i*. $c_{j}$ represents the capacity of community *j*. $p_{ij}$ represents the path between community *i* and shelter *j*. $x_{ij}$ is the decision-making variable and represents the number of evacuees allocated to path $p_{ij}$. Then, the corresponding evacuation time on this road is a convex function, represented by ${g_{ij}(x}_{ij})$. Since different numbers of allocated evacuees will correspond to different evacuation times, the function ${g_{ij}(x}_{ij})$ must be a one-to-one mapping. The model can be written as the following convex program:

(1) $$\begin{array}{c} \min_{g}\max_{i,j}\left\{g_{ij}\left(x_{ij}\right)\right\} \end{array}$$

(2) $$\begin{array}{ccc} \sum_{j=1}^{n}x_{ij} & = & b_{i},i=1,2,\cdots ,m; \end{array}$$

(3) $$\begin{array}{ccc} \sum_{i=1}^{m}x_{ij} & \leq & c_{j},j=1,2,\cdots ,n; \end{array}$$

(4) $$\begin{array}{ccc} x_{i,j} & \geq & 0,x\in Z \end{array}$$

It is assumed that there is no mutual influence between any two roads; the traffic conditions on a road do not affect those of another. The problem can be described by bipartite graph $G=\left(I_{1}{,\;\;J}_{1}\right)$, in which the vertex set $I_{1}$ and the vertex set $J_{1}$ correspond to the community set *I* and the shelter set *J*, respectively. There is an edge connection between vertex ${i\in I}_{1}$ and vertex ${j\in J}_{1}$ only when there is a path between community *i* and shelter *j*.

### 3.2. Approximation Algorithm Based on Network Flow

#### 3.2.1. Network Flow Graph Structure Construction

**Figure 2:**
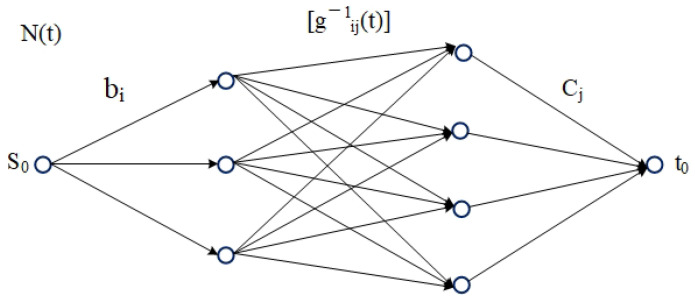
Network flow diagram $N\left(t\right)$.

The network flow graph is constructed on the bipartite graph $G=\left(I_{1}{,\;\;J}_{1}\right)$, as shown in Figure 2. For any given parameter *t*, vertex set $V=\left\{s_{0},t_{0},I,J\right\}$, where $s_{0}$ is the super-source point and $t_{0}$ is the over-closing point. The arc set A includes the following three types:

1. When $\left(s_{0},i\right),\forall i\in I,$ the corresponding capacity is $b_{i}$.
2. When $\left(j,t_{0}\right),\forall j\in J$, the corresponding capacity is $c_{j}$.
3. In the arc between *I* and *J*, the set of arcs whose time required for a single person *i* to *j* transfer to the network is less than the parameter *t* is incorporated into the network. That is, if $\forall i\in I,\;\forall j\in J,\left(i,j\right)\in A$ and only if $g_{ij}\left(1\right)\leq t$, then the capacity limit on this arc is $\left\lfloor g_{ij}^{-1}\left(t\right)\right\rfloor$, where $\left\lfloor g_{ij}^{-1}\left(t\right)\right\rfloor$ represents the inverse function of $g_{ij}$ and ⌊⌋ represents the same but rounded down. Since the function ${g_{ij}(x}_{ij})$ is a one-to-one mapping, its inverse function $g_{ij}^{-1}$ must exist.

When the problem is described as the network flow graph in Figure 2, once the functional relationship $g_{ij}\left(t\right)$ of the number of people to be evacuated and the time on each path is given, the inverse function can be used to find the capacity of each path under the limit of *t*; then, it constitutes a network flow graph with all parameters known. The maximum flow problem is a classical network flow problem. One may refer to Schrijver [30] for further details of this problem. The most popular algorithm for solving the maximum flow problem is the Ford–Fulkerson algorithm. When using this maximum flow algorithm on the graph to find the maximum flow value on the network, this maximum flow value is the maximum number of people who can evacuate in the network under parameter *t*. If the total number of people evacuated is equal to the total number of people to be evacuated, then everyone can be evacuated to a safe area within time *t*. However, we require the shortest evacuation time to evacuate all the crowd to the shelter, so t is not necessarily the shortest evacuation time. Next, we use a larger time limit T as the initial solution, take the middle value of [0, T], T/2, and calculate the maximum flow under this time limit. Then, according to the calculation results, the binary search method is used to search step by step, and the optimal evacuation time is gradually reduced to a small temporal interval. When the length of the interval is less than a certain small number ϵ, we obtain a nearly optimal evacuation time *t* with high precision, and correspondingly obtain a specific evacuation plan under the evacuation time *t*.

#### 3.2.2. Binary Search Algorithm Based on Network Flow

An approximate algorithm for this problem is given for the network flow graph. It should be emphasized that the algorithm proposed below does not directly calculate the number of people allocated to each path to minimize the evacuation time, but takes the parameter *t* on the network flow graph as the decision variable and a sufficiently large constant *T* as the initial value. By searching for the optimal evacuation time $t^{*}$ step by step, the maximum flow algorithm is used on the network flow graph $N\left(t^{*}\right)$ to obtain the number of evacuees allocated on each path.

**Theorem** **1.** The accuracy of Algorithm 1 can be $\frac{t}{t^{*}}\leq \left(1+\varepsilon \right)$, where $t^{*}$ refers to the optimal solution of the problem.

**Algorithm 1:** Binary search algorithm based on network flow

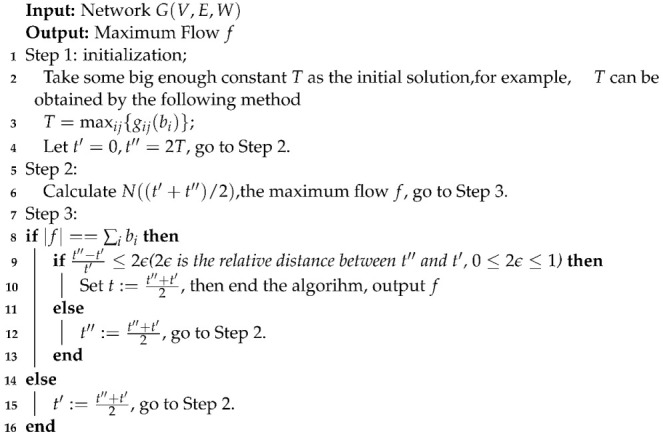

**Proof** **of** **Theorem** **1.** Suppose that the evacuation time on each path is a convex function of the number of evacuees allocated on the path [24]; that is, $g_{ij}^{-1}\left(t\right)$ is a convex function. Then, it is guaranteed that the local optimum found by *k* iterations is the global optimum. Assume that the time interval of the KTH iteration is $\left[t^{\prime },\;t^{\prime \prime }\right]$; then, $t=\frac{t^{\prime }+\;t^{\prime \prime }}{2}$, and since $t^{*}$ is in the interval of ${[t}^{\prime },\;t^{\prime \prime }]$, $t^{*}\geq t^{\prime }$.

Thus, the accuracy of Algorithm 1 is: $\frac{t}{t^{*}}=\frac{t^{\prime }+t^{\prime \prime }}{2t^{*}}.$ Else, because $\frac{t^{\prime \prime }-t^{\prime }}{t^{\prime }}\leq 2\varepsilon$, $t^{\prime \prime }\leq 2\varepsilon t^{\prime }+t^{\prime },$ $\frac{t}{t^{*}}=\frac{t^{\prime }+t^{\prime \prime }}{2t^{*}}\leq \frac{2\varepsilon t^{\prime }+t^{\prime }+t^{\prime }}{2t^{\prime }}=1+\varepsilon .$ □

**Theorem** **2.** *The complexity of Algorithm 1 is:*

$$O\left(\log\left(\frac{T}{\varepsilon }\right)\cdot \frac{n^{3}+n}{4}\right).$$

**Proof** **of** **Theorem** **2.**  For each iteration in Algorithm 1, the length of the interval of $\left[t^{\prime },\;t^{\prime \prime }\right]$ is reduced to half of the previous iteration. Then, on the network $N(\frac{t^{\prime }+t^{\prime \prime }}{2})$, the labeling algorithm is used to calculate the maximum flow value on the network.

The complexity of the labeling algorithm is $\frac{n^{3}+n}{4}$ [31], so the complexity generated by each iteration is $\frac{n^{3}+n}{4}$.

Starting from the initial value *T*, it is assumed that after *k* iterations of the algorithm, precision of parameter *t* can be achieved as $\frac{t}{t^{*}}\leq \left(1+\varepsilon \right),$ namely, $t=O\left(\left(1+\varepsilon \right)\cdot t^{*}\right)=O\left(\varepsilon \cdot t^{*}\right);$ then, $\frac{T}{2^{k}}=O\left(\varepsilon \cdot t^{*}\right).$

When the number of iterations *k* is:

$$k=O\left(\log\left(\frac{T}{{\varepsilon \cdot t}^{*}}\right)\right)=O\left(\log\left(\frac{T}{\varepsilon }\right)\right).$$

As *n* is the number of vertices on the directed graph G, the complexity of Algorithm 1 can be obtained as:

$$O\left(\log\left(\frac{T}{\varepsilon }\right)\cdot \frac{n^{3}+n}{4}\right)$$

□

#### 3.2.3. Theoretical Comparison

In this paper, a nonlinear model is established, and a polynomial time approximation algorithm is given, assuming no crossover between different paths and considering the nonlinear relationship between crowd density and evacuation speed. The complexity and accuracy of the algorithm are proved by theory, and the method’s effectiveness is verified by an example. The classical evacuation model only needs to satisfy the capacity limitation of road and shelter, but the relationship between evacuation time and evacuation number is a nonlinear convex function [32]. The innovative point of this paper is to propose an approximate algorithm with very good complexity and precision, which provides an effective solution for a nonlinear model of evacuation path planning.

Compared to the existing network flow algorithm that generally can only solve the linear programming problem, we consider the nonlinear evacuation time with respect to the crowd density in the model, and first formulate the problem as a nonlinear network flow model. It is a binary search model based on the classical maximum flow algorithm (namely, the Ford—Fulkerson algorithm) and the accuracy ratio is guaranteed to be within a pre-defined number (say, 0.05%). The existing methods in the literature either are unable to guarantee the accuracy ratio or are unable to deal with the nonlinear function in the model.

Theoretically, this research proves the accuracy of the algorithm to be within any pre-defined small number in Theorem 1, and we also prove the complexity of the algorithm is polynomial in Theorem 2. These two theorems have shown the algorithm has high accuracy and polonomial computational complexity.

## 4. Empirical Study of the Evacuation Route Planning Problem

### Experiment Description

**Figure 3:**
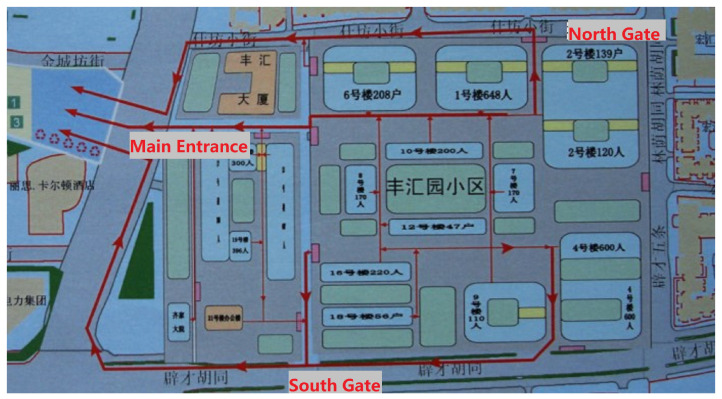
Building map and exit marker. The blue rectangular area indicates the building number of the evacuees and red lines indicate evacuation routes.

Take the earthquake evacuation of the local community as an example. There are 16 buildings in this community; and there are three exits, namely, the north gate, the main entrance, and the south gate, as shown in Figure 3. The population of the community is divided into three parts and evacuated through the three gates, as shown in Table 1.

There are seven open shelters on Financial Street, and the capacity of each shelter is shown in Table 2.

The functional relationship between evacuation speed and density adopts the functional relationship between evacuation speed and crowd density proposed by Pursals [24], as follows:

(5) $$v\left(\rho_{ij}\right)=\left\{\begin{array}{c} 0.8568\lambda_{ij},\;0<\rho_{ij}\leq 0.5382\\ \left(1-0.266\rho_{ij}\right)\lambda_{ij},\;0.5382<\rho_{ij}\leq 3.5 \end{array}\right.$$

where *v* is the crowd evacuation speed and $\lambda_{ij}$ is the average walking speed on the road $P_{ij}$, both in unit m/s. $\rho_{ij}$ is the crowd density able to enter the road in person/m^2^. Formula (1) is converted into a functional relationship between time and crowd number:

(6) $$t_{ij}\left(x_{ij}\right)=\left\{\begin{array}{c} \frac{1}{0.8568\lambda_{ij}}\left[l_{ij}+\frac{a_{ij}}{w_{ij}}\right],\;0<x_{ij}<x_{ij,I}\\ \frac{a_{ij}}{\left(a_{ij}-0.266x_{ij}\right)\lambda_{ij}}\left[l_{ij}+\frac{a_{ij}}{w_{ij}}\right],\;x_{ij,I}<x_{ij}<x_{ij,S} \end{array}\right.$$

**Figure 4:**
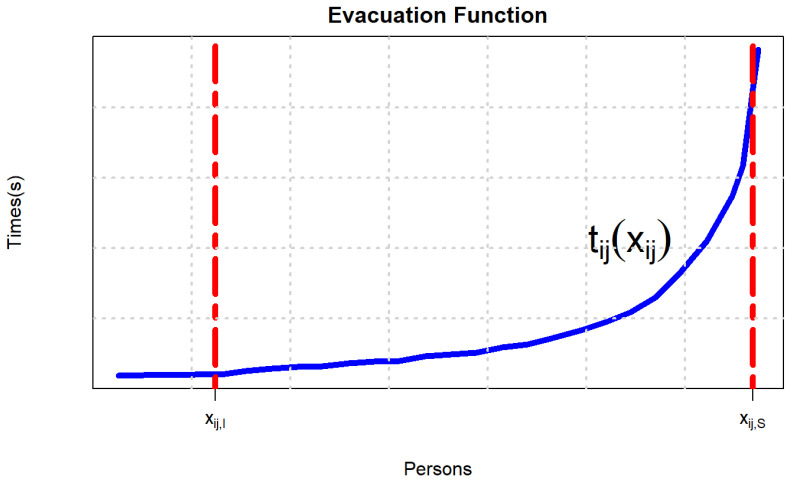
Function diagram of evacuation time and the number of evacuees.

$x_{ij,I}=0.5382a_{ij}$ and $x_{ij,S}=3.5a_{ij}$ refer to the lower and upper bounds of the number of evacuees allocated to the road $P_{ij}$, respectively. $t_{ij}\left(x_{ij}\right)$ represents the time taking to allocate the number of $x_{ij}$ people on the path $P_{ij}$. $l_{ij}$ represents the length of the road $P_{ij}$. $w_{ij}$ is the width of the road $P_{ij}$. $a_{ij}$ represents the occupied area of evacuees allocated to road $P_{ij}$ when they reach the intersection of $P_{ij}$. $\lambda_{ij}$ represents the normal walking speed on $P_{ij}$. When $\lambda_{ij}$ = 1.4 m/s, the analysis shows that the evacuation time increases with the increase in number of people, so the relationship between the evacuation time and the number of people evacuated is a convex function, as shown in Figure 4.

**Figure 5:**
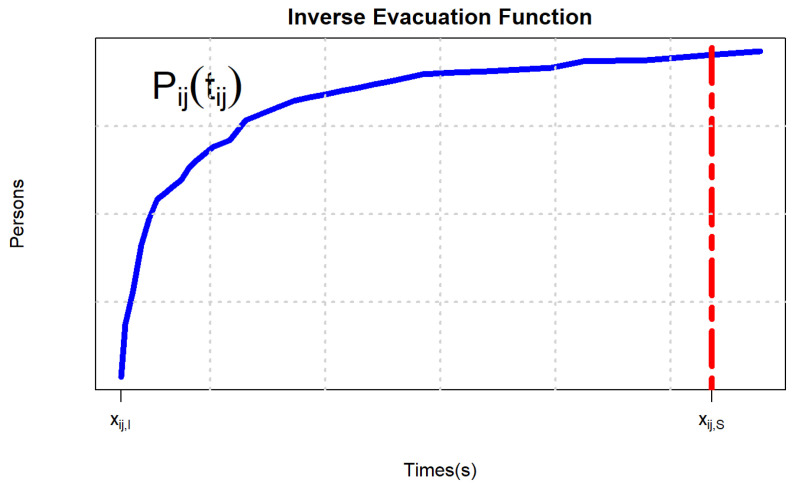
Function diagram of evacuation number and evacuation time.

The inverse function of $t_{ij}\left(x_{ij}\right)$ is the function of the evacuation time on a path and the number of evacuees assigned to the path.

(7) $$P_{ij}\left(t_{ij}\right)=\left\{\begin{array}{c} t_{ij}^{-1}\left(x_{ij}\right)=0,\;0\leq t_{ij}<t_{ij,I}\\ x_{ij,I},\;t_{ij}=t_{ij,I}\\ \frac{a_{ij}}{0.266}\left(1-\frac{l_{ij}w_{ij}+a_{ij}}{x_{ij}\lambda_{ij}w_{ij}}\right),\;t_{ij,I}<t_{ij}\leq t_{ij,S} \end{array}\right.$$

where $t_{ij,I}$ and $t_{ij,S}$ refer to the lengths of time required for the evacuation of a certain number of people at densities of 0.5382 person/m^2^ and 3.5 person/m^2^ to the destination, as shown in Figure 5, which is a concave function. In addition, parameter values of each path, including road length, road width, and intersection area, are measured using Google Maps, as shown in Table 3. There are 21 paths from 3 evacuation points to 7 shelters.

## 5. Computational Results of the Empirical Study

Algorithm 1 was simulated using Matlab software. The algorithm’s accuracy was set to be less than 0.05%, i.e., ε=0.05%. We calculated the optimal evacuation scheme of the above example, including evacuation allocated on each path, crowd evacuation speed, and time. The results are shown in Table 4.

The total evacuation time and algorithm accuracy are shown in Table 5.

**Figure 6:**
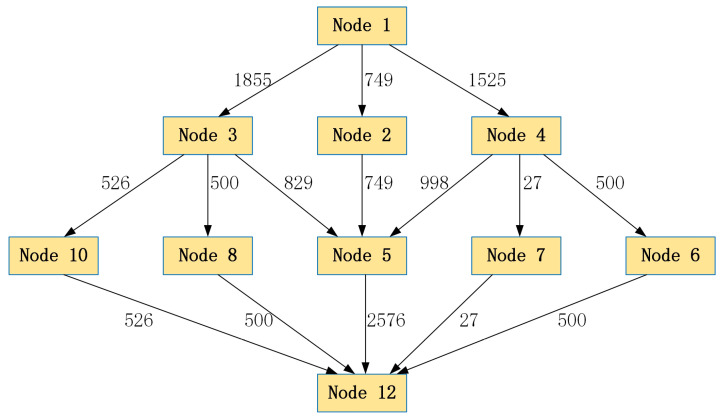
Network flow diagram of community evacuation path planning calculated by Algorithm 1.

The results show that the accuracy of the optimal target value obtained by the algorithm reached 0.0005 over 13 iterative operations. The total evacuation time was around 24.92 min. The upper bound and lower bound in the 13th iteration had a gap of 0.041%. This indicates that the optimal evacuation time located in between the upper and lower bounds must be within 0.04% deviation from the optimal value, which is lower than the predefined accuracy level of 0.005. Finally, the network flow diagram obtained is shown in Figure 6.

### Analysis of Results and Discussion

In this empirical study of the Fenghuiyuan community, we used Algorithm 1 and obtained the routing results in seconds. The accuracy of the obtained evacuation results reached 0.05%. The theoretical and practical results show that the algorithm has four advantages. Firstly, the algorithm is simple, intuitive, and easy to implement in programming languages. Secondly, an effective method for solving nonlinear evacuation problems is provided, with high precision, low complexity, and fast calculation speed. Thirdly, no matter how complex the specific nonlinear function relationship between the number of evacuated people and the evacuation time, as long as the function is invertible, the algorithm can be used to solve it. Therefore, the scope of application is wide. Fourthly, the precision is high, and precision of any preset value can be achieved.

The results in Table 4 and Figure 6 show the detailed evacuation plan for the earthquake emergencies of this empirical study. We found that 4129 people were evacuated in 24.92 min, which is an executable plan for emergencies. In this evacuation plan, six paths were used in the network. The maximum evacuation flow occurred on path (4,5) with 998 persons, while the minimum flow bottleneck was on path (4,7) with only 27 persons. Thus, we might need to improve the capacity on path (4,7) so that more flow can be shared on this path to reduce flow on other paths. Correspondingly, the evacuation time could be reduced. Using Algorithm 1, we can quickly obtain the evacuation plan for a community and provide suggestions for improving the plan’s efficiency, making it a valuable tool for the decision making of relevant authorities or managers.

## 6. Conclusions

This paper studied the nonlinear model construction of intelligent route planning in community evacuation. The nonlinear model reflects the nonlinear relationship between the evacuation time and the number of people evacuated; that is, as the number of people increases, the evacuation speed will slow down, and the evacuation time will increase. In order to solve the nonlinear model of evacuation route planning in the community evacuation problem, we proposed a binary search method based on the maximum flow algorithm within the network flow method to solve the nonlinear model. Finally, we empirically studied Fenghuiyuan community’s earthquake evacuation. Our proposed algorithm provides efficient and highly accurate results.

In a nutshell, the algorithm designed in this paper provides an ingenious idea for solving evacuation problems, especially nonlinear evacuation problems. Avoiding starting from the problem itself, the algorithm starts indirectly, taking the evacuation time as the solution variable. By searching for the optimal evacuation time step by step, the maximum flow algorithm finds the number of evacuees allocated on each path under the determined evacuation time.

However, the proposed algorithm has some limitations. It is currently only applicable for the situation where there is no intersection between the evacuation paths. For the situation where the roads intersect, the complexity of the model and algorithm will be increased. Therefore, in cases of more complex road conditions, the algorithm needs to be further expanded and further analyzed.

## Figures and Tables

**Table 1:** Evacuation exit allocation table.

Exit	Building No.	Total Number of People
The north gate	1, 2, 6	749
Main entrance	7, 8, 12, 13, 15, 17	1855
The south gate	4, 9, 10, 16, 18, 19, 21	1525

**Table 2:** Statistical table of shelters in open spaces.

No.	Location	Available Area (m^2^)	Capacity (2 m^2^/Person)
1	The green belt on WSK Road	10,000	5000
2	Shiyan Middle School	1000	500
3	Lu Xun Secondary School	1000	500
4	Fendou Primary School	1000	500
5	No. 159 Middle School	2000	1000
6	Jiexin Park, Financial Street	10,000	5000
7	Chenghuang Temple	1000	500

**Table 3:** Parameter values of each evacuation path.

Path $p_{\mathrm{ij}}$	Length: $l_{\mathrm{ij}}$ (km)	Width: $w_{\mathrm{ij}}$ (m)	Available Area: $a_{\mathrm{ij}}$ (m^2^)
*i* = 1, *j* = 1	1.4	4	450
*i* = 1, *j* = 2	1.35	4	450
*i* = 1, *j* = 3	1.6	4	450
*i* = 1, *j* = 4	1.1	4	450
*i* = 1, *j* = 5	0.85	4	450
*i* = 1, *j* = 6	1.1	4	450
*i* = 1, *j* = 7	2	4	450
*i* = 2, *j* = 1	1.9	5	750
*i* = 2, *j* = 2	2	5	750
*i* = 2, *j* = 3	2.1	5	750
*i* = 2, *j* = 4	1.9	5	750
*i* = 2, *j* = 5	2.3	5	750
*i* = 2, *j* = 6	1.5	5	750
*i* = 2, *j* = 7	1.4	5	750
*i* = 3, *j* = 1	1.5	4	600
*i* = 3, *j* = 2	1.4	4	600
*i* = 3, *j* = 3	1.1	4	600
*i* = 3, *j* = 4	1.5	4	600
*i* = 3, *j* = 5	1.5	4	600
*i* = 3, *j* = 6	1.2	4	600
*i* = 3, *j* = 7	1.6	4	600

**Table 4:** Algorithm 1 calculation results.

No.	Path: $p_{\mathrm{ij}}^{\prime }$	Toll: $x_{\mathrm{ij}}$	Speed: $v_{\mathrm{ij}}$ (m/s)	Time: $t_{\mathrm{ij}}$ (s)
1	(2, 5)	749	0.8144	7574.0769
2	(3, 5)	829	0.8013	12,734.4399
3	(4, 5)	998	1.1766	6565.7510
4	(4, 6)	500	0.7213	12,810.9734
5	(4, 7)	27	1.1995	11,871.4152
6	(3, 8)	500	0.7523	10,290.5930
7	(3, 10)	526	1.1620	12,723.9683

**Table 5:** Algorithm 1 calculation effects and target values.

Total Evacuation Time	t=1495.5s≈24.92 min
Upper and lower bounds of last iteration $\left[t^{\prime },\;t^{\prime \prime }\right]$	[1495.18, 1495.79]
The number of iterations	k=13
Results in the accuracy	ε=0.0005

## Data Availability

The data presented in this study are available on request from the corresponding author. The data are not publicly available due to privacy reasons.
